# Supplementary figures and images for: The AMA1-RON complex drives Plasmodium sporozoite invasion in the mosquito and mammalian hosts
Source: PLoS Pathog. 2022 Jun 22;18(6):e1010643. doi: 10.1371/journal.ppat.1010643 (PMC9255738; doi:10.1371/journal.ppat.1010643)

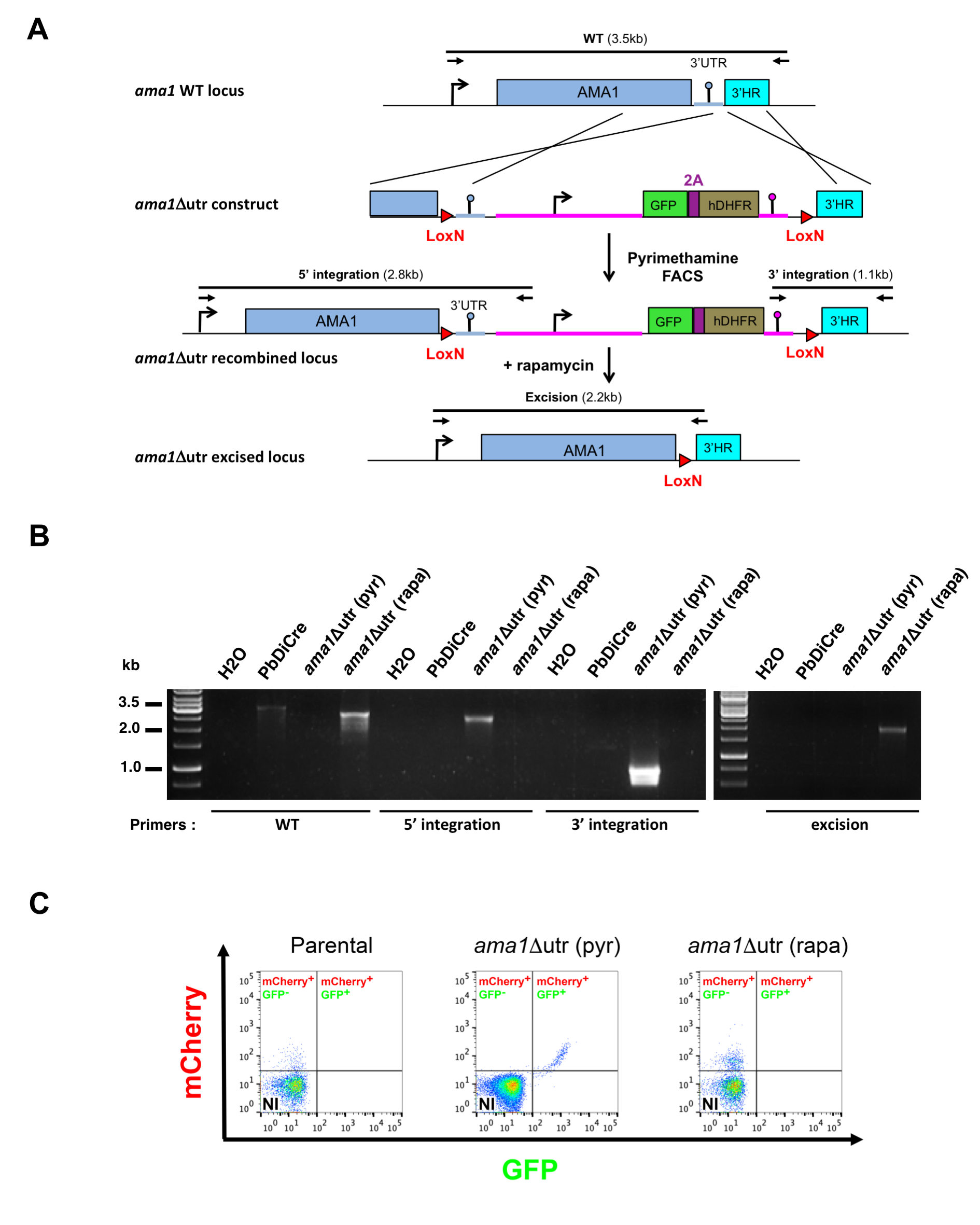

Supplement: S1 Fig — A. Strategy to generate ama1Δutr parasites. The wild-type locus of P. berghei ama1 in the PbDiCre parasite line was targeted with a ama1Δutr replacement plasmid containing 2 Lox sites and 5’ and 3’ homologous sequences inserted on each side of a GFP-2A-hDHFR cassette. Upon double crossover recombination, the LoxN sites are inserted upstream of the 3’ UTR and downstream of the GFP-2A-hDHFR cassette, respectively. Activation of the DiCre recombinase with rapamycin results in excision of the 3’ UTR together with the GFP-2A-hDHFR cassette. Genotyping primers and expected PCR fragments are indicated by arrows and lines, respectively. B. Genotyping of parental PbDiCre and ama1Δutr transfected parasites after pyrimethamine selection (pyr) and after rapamycin treatment (rapa) of the final population. Parasite genomic DNA was analyzed by PCR using primer combinations specific for the unmodified locus (WT), the 5’ integration, 3’ integration or excision events. C. Flow cytometry analysis of PbDiCre (parental) and ama1Δutr blood stage parasites after pyrimethamine selection (pyr) or rapamycin exposure (rapa). NI, non-infected red blood cells. (TIF) [file ppat.1010643.s004.tif]

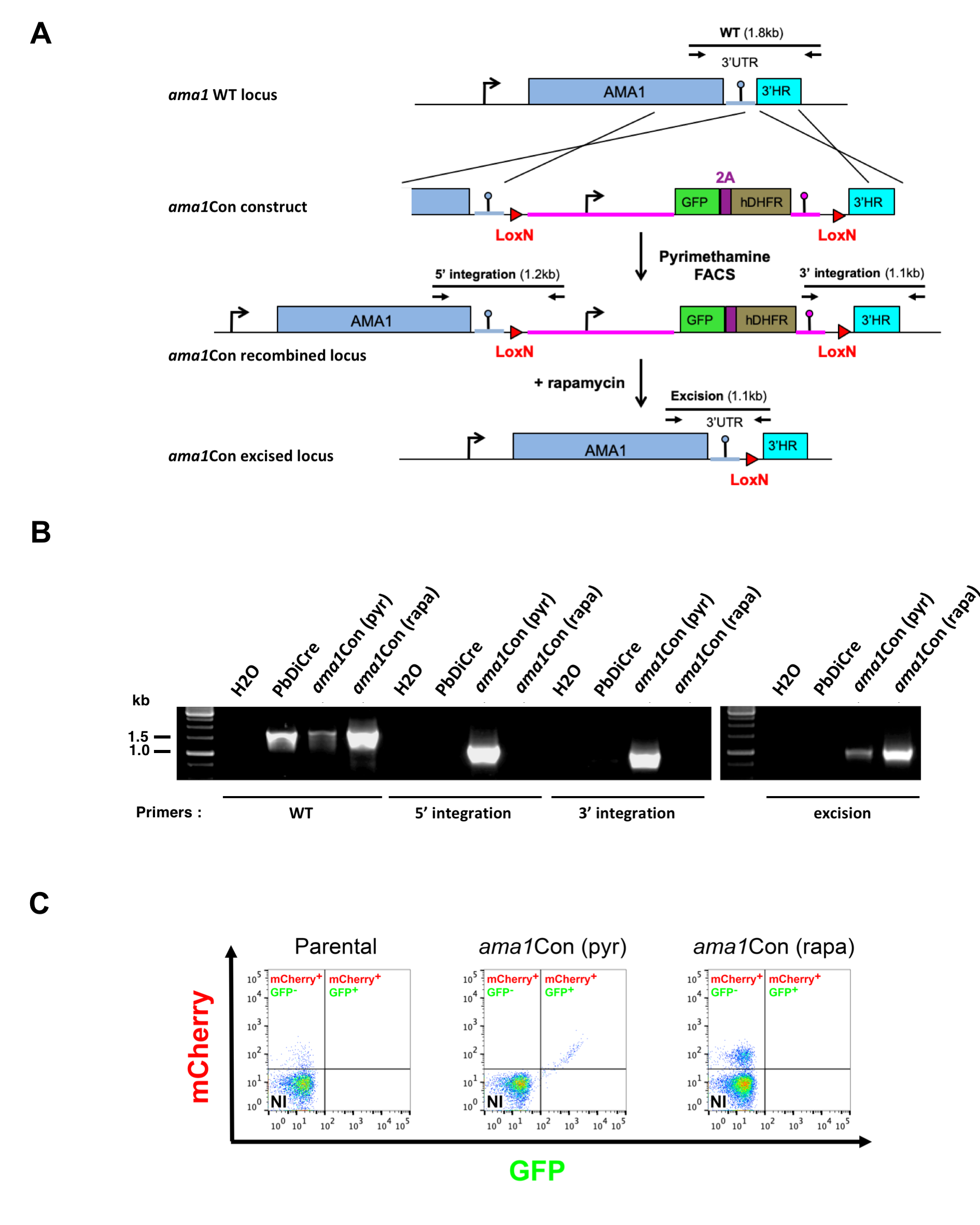

Supplement: S2 Fig — A. Strategy to generate ama1Con parasites. The construct is similar to the ama1Δutr construct, except that the first LoxN site is located downstream of the 3’ UTR. Upon rapamycin-induced excision, the ama1 locus remains intact. B. Genotyping of parental PbDiCre and ama1Con transfected parasites after pyrimethamine selection (pyr) and after rapamycin treatment (rapa) of the final population. Parasite genomic DNA was analyzed by PCR using primer combinations specific for the unmodified locus (WT), the 5’ integration, 3’ integration or excision events. C. Flow cytometry analysis of PbDiCre (parental) and ama1Con blood stage parasites after pyrimethamine selection (pyr) or rapamycin exposure (rapa). NI, non-infected red blood cells. (TIF) [file ppat.1010643.s005.tif]

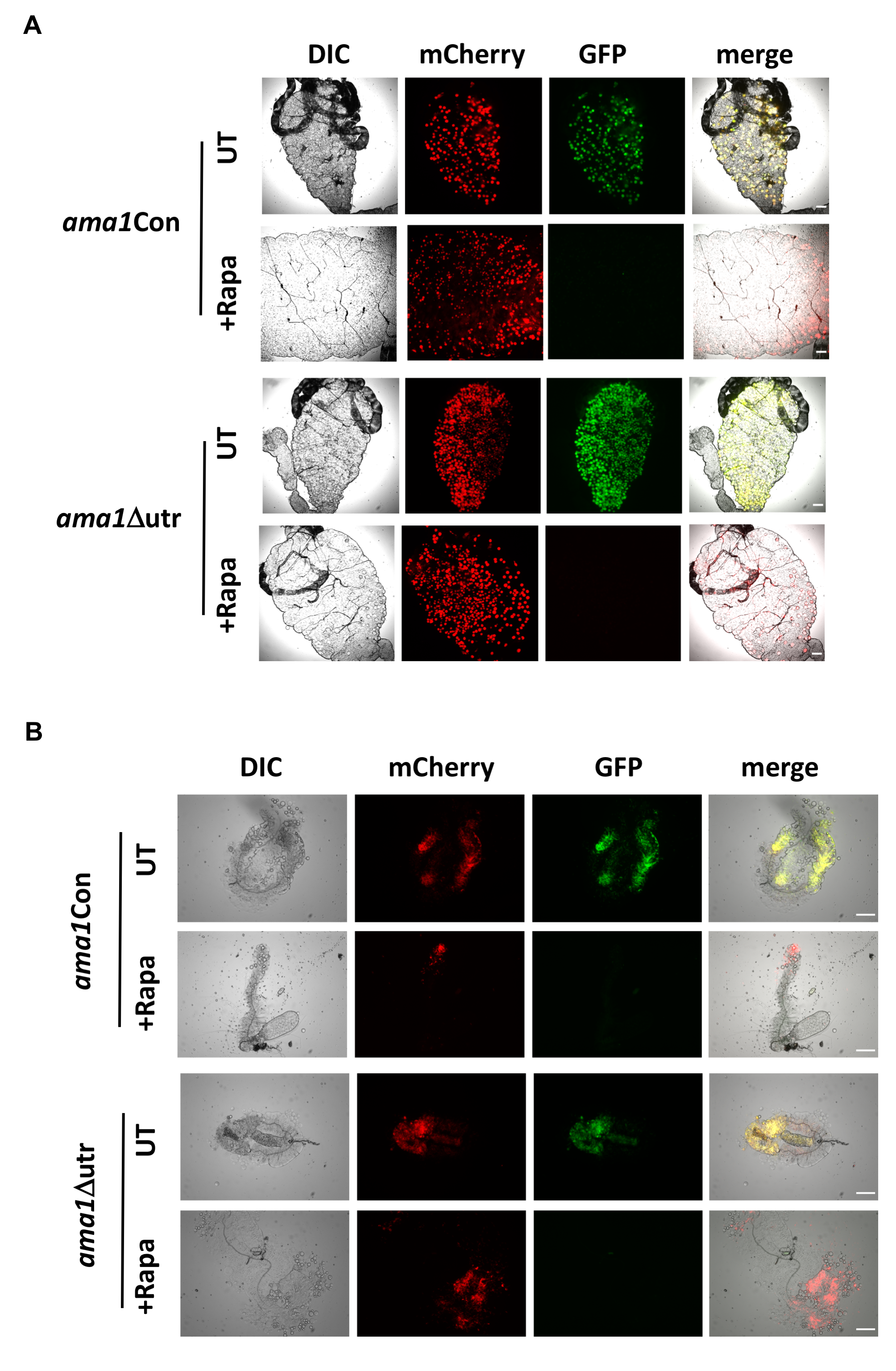

Supplement: S3 Fig — A. Fluorescence microscopy images of midguts from mosquitoes infected with untreated (UT) or rapamycin-treated (rapa) ama1Con and ama1Δutr parasites. Scale bar = 200 μm. B. Fluorescence microscopy images of salivary glands from mosquitoes infected with untreated (UT) or rapamycin-treated (rapa) ama1Con and ama1Δutr parasites. Scale bar = 200 μm. (TIF) [file ppat.1010643.s006.tif]

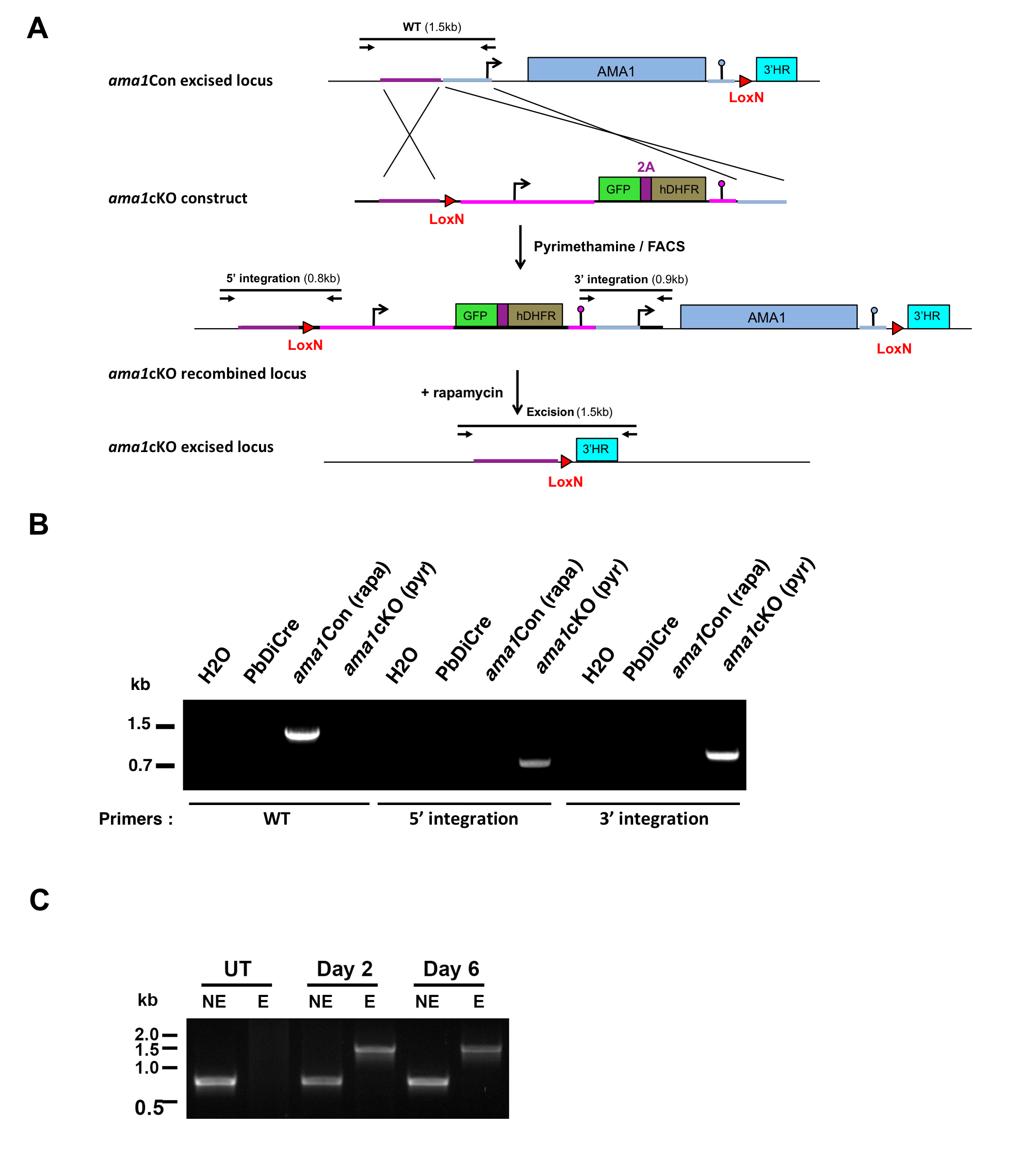

Supplement: S4 Fig — A. Strategy to generate ama1cKO parasites. The ama1 locus in rapamycin-treated (excised) ama1Con parasites was targeted with a ama1cKO replacement plasmid containing a single LoxN site and 5’ and 3’ homologous sequences inserted on each side of a GFP-2A-hDHFR cassette. Upon double crossover recombination, a second LoxN site is inserted upstream of the GFP-2A-hDHFR cassette and ama1 gene. Activation of the DiCre recombinase with rapamycin results in excision of the entire ama1 gene together with the GFP-2A-hDHFR cassette. Genotyping primers and expected PCR fragments are indicated by arrows and lines, respectively. B. Genotyping of PbDiCre, rapamycin-treated (excised) ama1Con (parental) and ama1cKO parasites after selection with pyrimethamine (pyr). Parasite genomic DNA was analyzed by PCR using primer combinations specific for the unmodified locus (WT), the 5’ integration and 3’ integration events. C. Genotyping of ama1cKO blood stage parasites collected 2 or 6 days after rapamycin exposure or left untreated (UT). Parasite genomic DNA was analyzed by PCR using primer combinations specific for the non-excised (NE, 5’ integration combination) or excised (E) locus. (TIF) [file ppat.1010643.s007.tif]

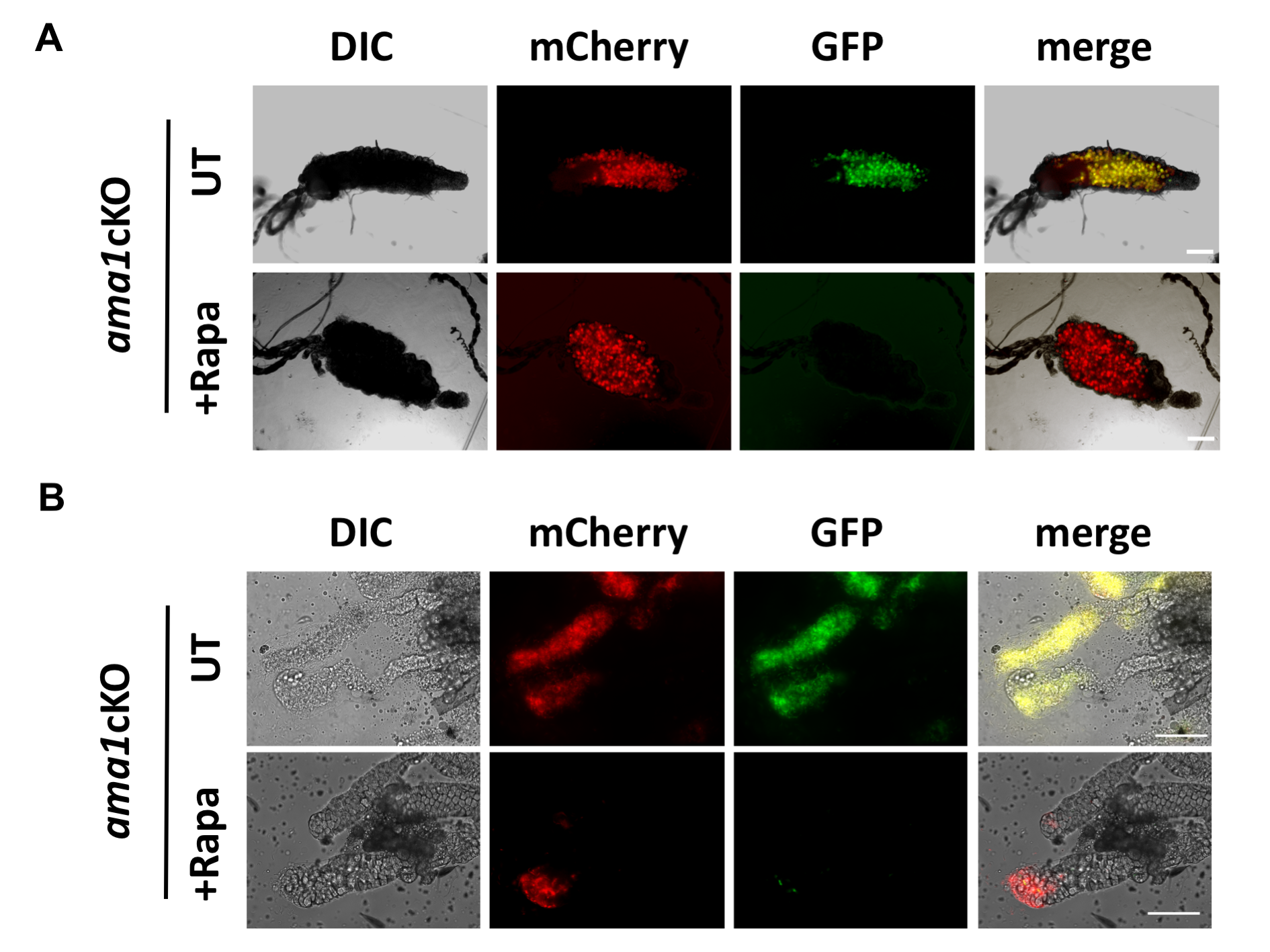

Supplement: S5 Fig — A. Fluorescence microscopy of midguts from mosquitoes infected with untreated (UT) or rapamycin-treated (rapa) ama1cKO parasites. Scale bar = 200 μm. B. Fluorescence microscopy of salivary glands isolated from mosquitoes infected with untreated (UT) or rapamycin-treated (rapa) ama1cKO parasites. Scale bar = 200 μm. (TIF) [file ppat.1010643.s008.tif]

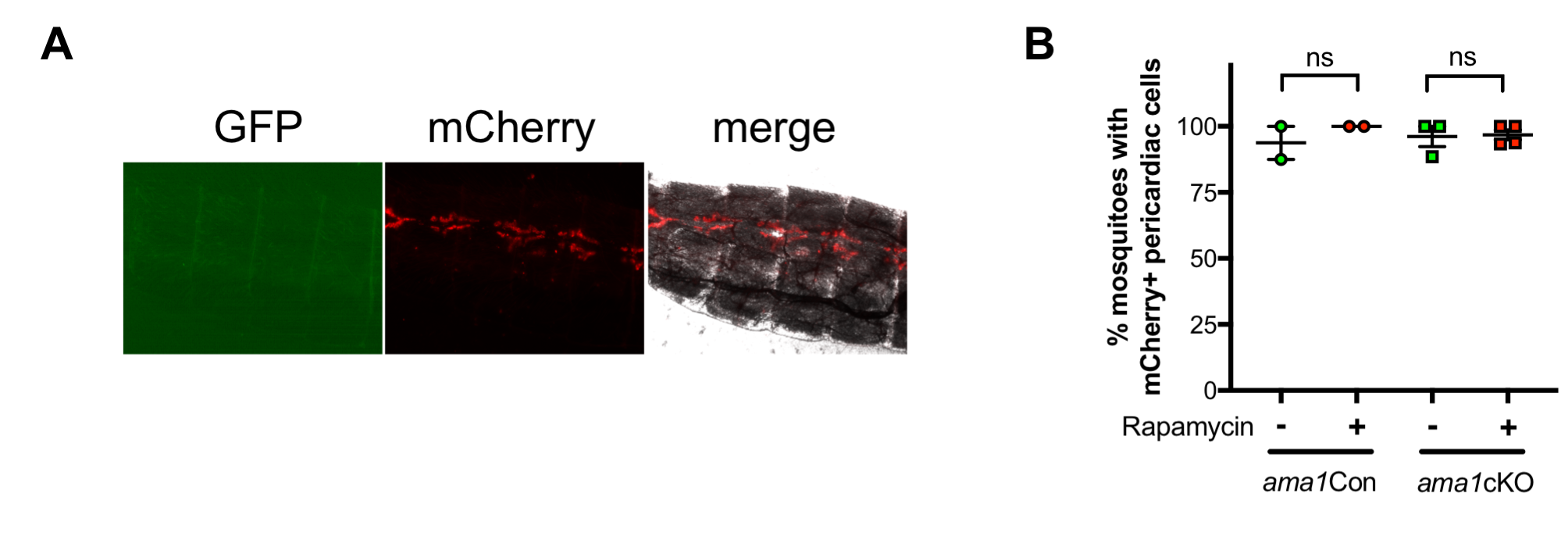

Supplement: S6 Fig — A. Imaging of the abdomen of a mosquito infected with rapamycin treated ama1cKO parasites, after removal of the midgut, showing mCherry-labelled pericardial structures. B. Quantification of mosquitoes with mCherry-labelled pericardial cells at D21 post-infection with untreated (UT) or rapamycin-treated (rapa) ama1Con and ama1cKO parasites. Ns, non-significant (Two-tailed ratio paired t test). (TIF) [file ppat.1010643.s009.tif]

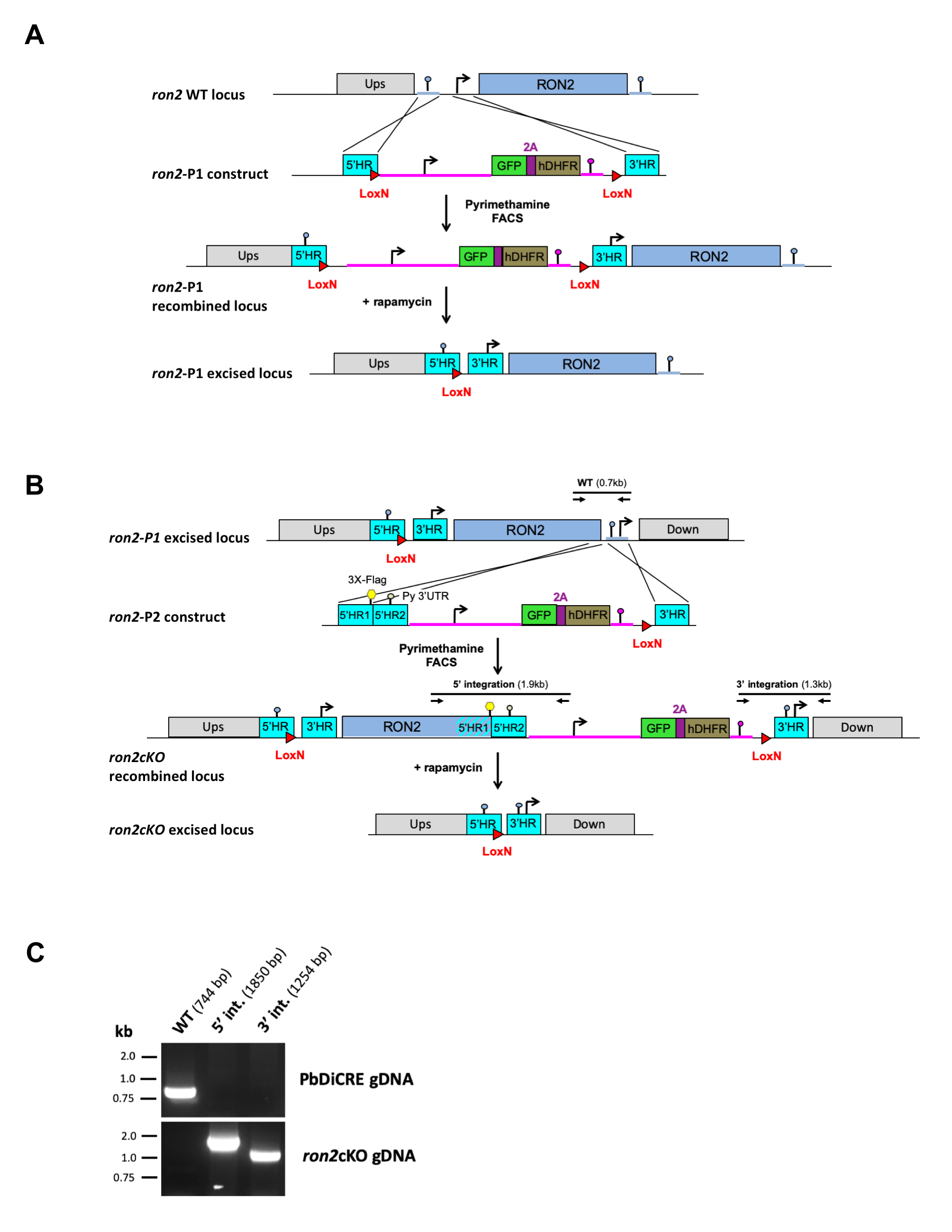

Supplement: S7 Fig — A-B. Two-step strategy to generate ron2cKO parasites. In the first step (A), the ron2 locus in PbDiCre parasites was targeted with a ron2-P1 replacement plasmid containing 5’ and 3’ homologous sequences and two LoxN sites flanking a GFP-2A- hDHFR cassette. Upon double crossover recombination, the two LoxN sites are inserted upstream of ron2. Activation of the DiCre recombinase with rapamycin results in excision of the GFP-2A-hDHFR cassette, leaving a single LoxN site upstream of the gene in excised ron2-P1 parasites. In the second step (B), the ron2 locus in rapamycin-treated (excised) ron2-P1 parasites was targeted with a ron2-P2 replacement plasmid containing 5’ and 3’ homologous sequences flanking a GFP-2A- hDHFR cassette and a single LoxN site. Upon double crossover recombination, the LoxN site is inserted downstream of ron2 and the GFP-2A- hDHFR cassette. Activation of the DiCre recombinase with rapamycin results in excision of the entire ron2 gene together with the GFP-2A-hDHFR cassette. Genotyping primers and expected PCR fragments are indicated by arrows and lines, respectively. C. Genotyping of PbDiCre and ron2cKO parasites. Parasite genomic DNA was analyzed by PCR using primer combinations specific for the unmodified locus (WT), the 5’ and 3’ integration events. (TIF) [file ppat.1010643.s010.tif]

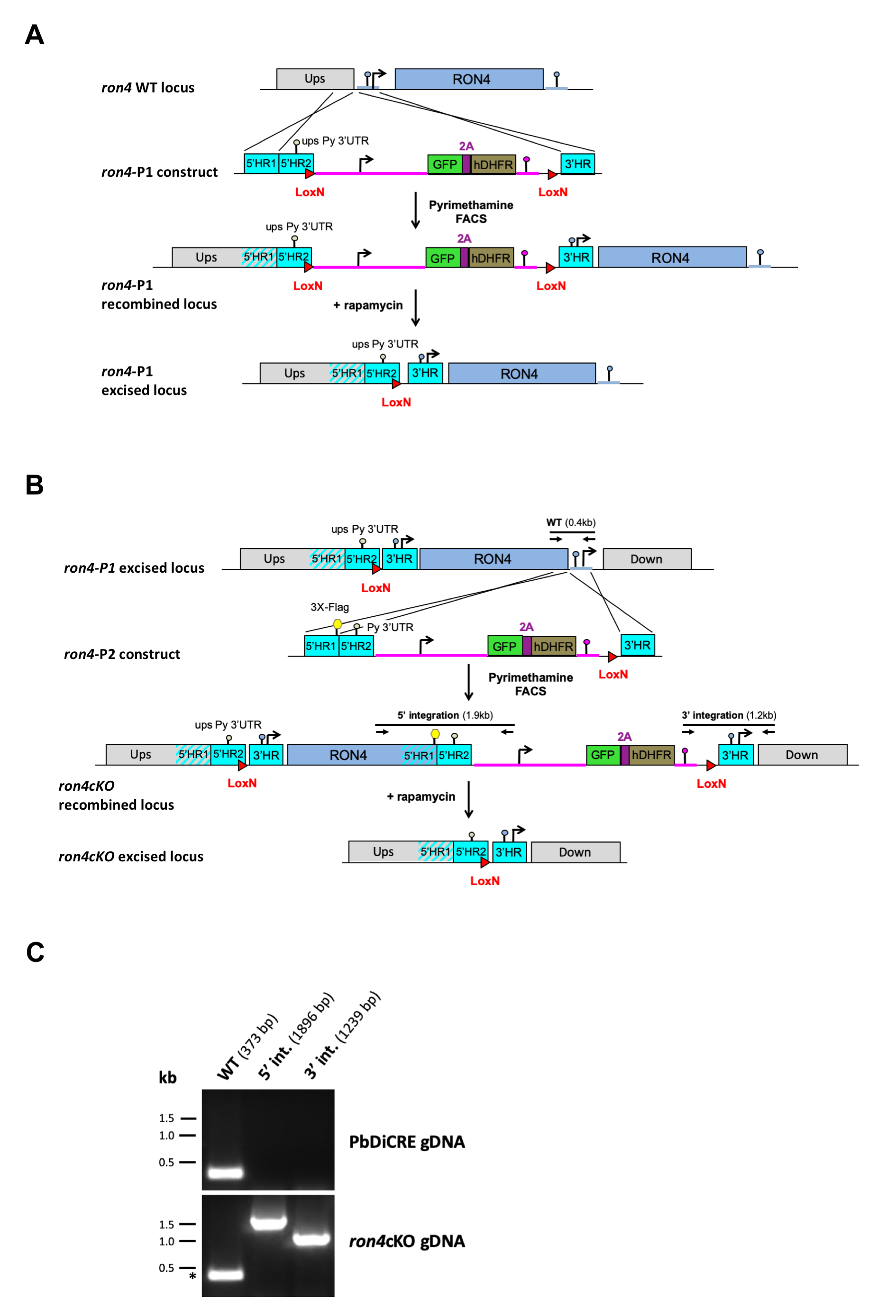

Supplement: S8 Fig — A-B. Two-step strategy to generate ron4cKO parasites. In the first step (A), the ron4 locus in PbDiCre parasites was targeted with a ron2-P1 replacement plasmid containing 5’ and 3’ homologous sequences and two LoxN sites flanking a GFP-2A- hDHFR cassette. Upon double crossover recombination, the two LoxN sites are inserted upstream of ron4. Activation of the DiCre recombinase with rapamycin results in excision of the GFP-2A-hDHFR cassette, leaving a single LoxN site upstream of the gene in excised ron4-P1 parasites. In the second step (B), the ron4 locus in rapamycin-treated (excised) ron4-P1 parasites was targeted with a ron4-P2 replacement plasmid containing 5’ and 3’ homologous sequences flanking a GFP-2A- hDHFR cassette and a single LoxN site. Upon double crossover recombination, the LoxN site is inserted downstream of ron4 and the GFP-2A- hDHFR cassette. Activation of the DiCre recombinase with rapamycin results in excision of the entire ron4 gene together with the GFP-2A-hDHFR cassette. Genotyping primers and expected PCR fragments are indicated by arrows and lines, respectively. C. Genotyping of PbDiCre and ron4cKO parasites. Parasite genomic DNA was analyzed by PCR using primer combinations specific for the unmodified locus (WT), the 5’ and 3’ integration events. (TIF) [file ppat.1010643.s011.tif]

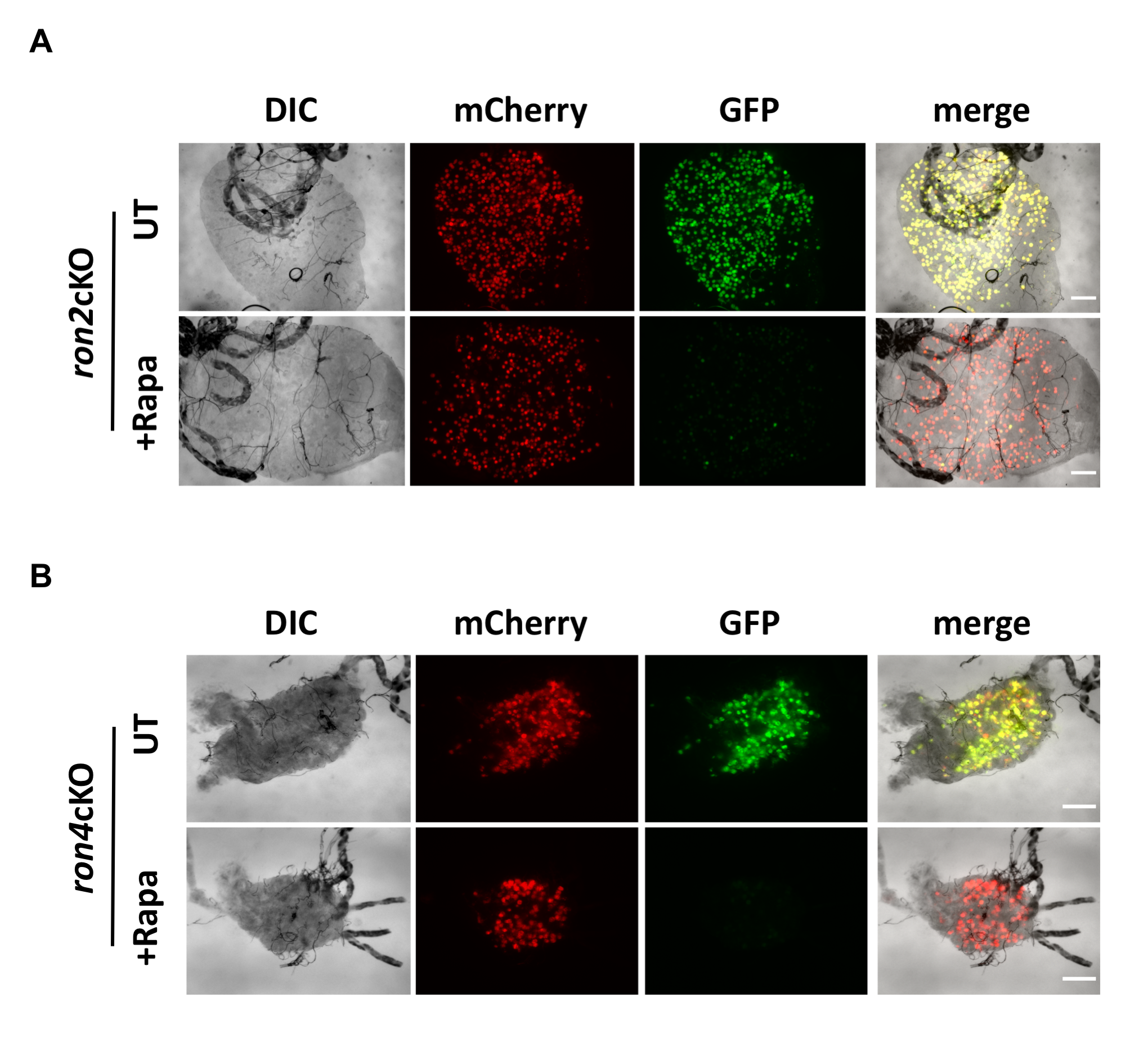

Supplement: S9 Fig — A-B. Fluorescence microscopy of midguts from mosquitoes infected with untreated (UT) or rapamycin-treated (rapa) ron2cKO (A) or ron4cKO (B) parasites. Scale bar = 200 μm. (TIF) [file ppat.1010643.s012.tif]

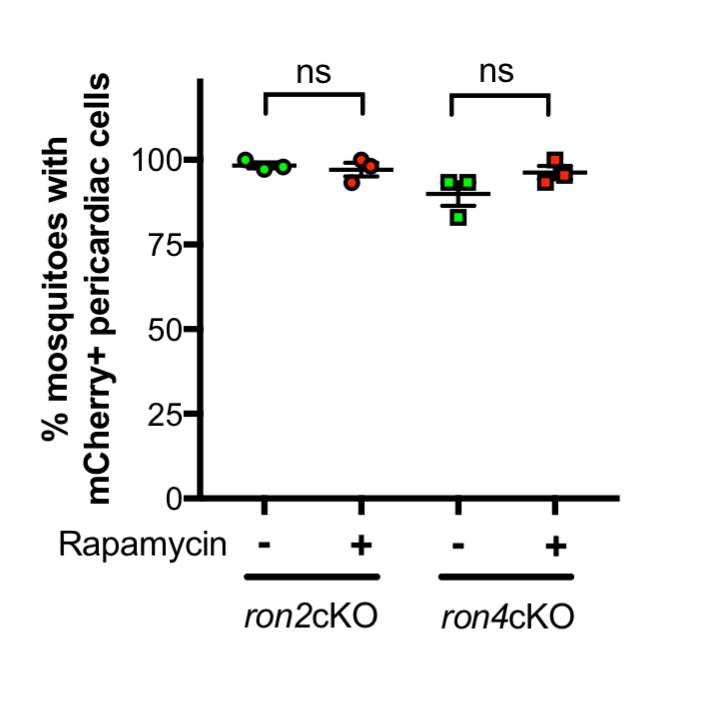

Supplement: S10 Fig — Quantification of mosquitoes with mCherry-labelled pericardial cells at D21 post-infection with untreated (UT) or rapamycin-treated (rapa) ron2cKO or ron4cKO parasites. Ns, non-significant (Two-tailed ratio paired t test). (TIF) [file ppat.1010643.s013.tif]

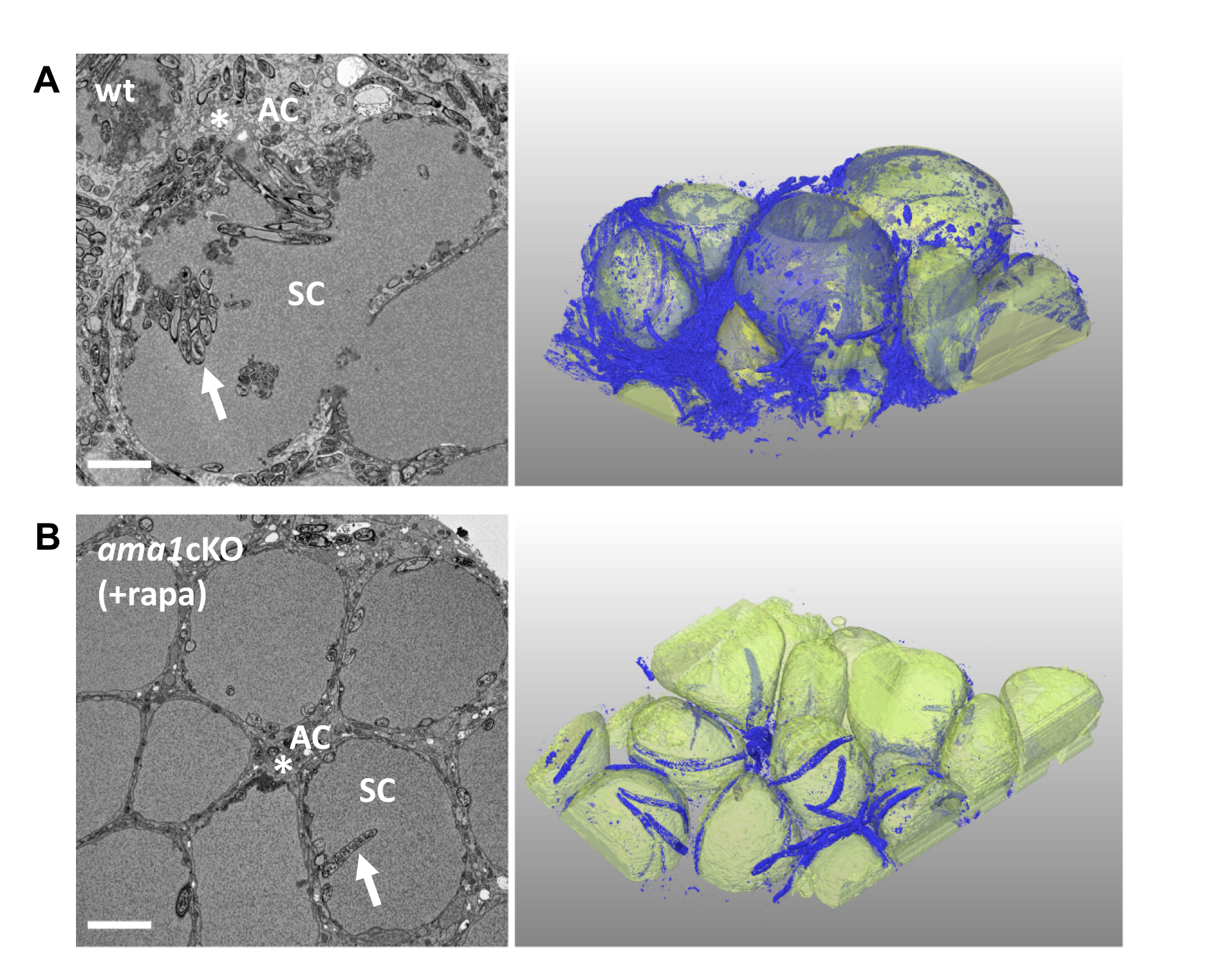

Supplement: S11 Fig — A-B. Representative sections of salivary glands from mosquitoes infected with WT (A) or rapamycin-treated ama1cKO (B) parasites (left panels). Scale bars, 5 μm. WT and AMA1-deficient sporozoites were observed inside the acinar cells (AC, asterisks) and in the secretory cavities (SC, arrows). The volume segmentation images (right panels) show the secretory cavities (yellow) and sporozoites (blue), and correspond to S1 Movie and S2 Movie, respectively, for WT and ama1cKO parasites. (TIF) [file ppat.1010643.s014.tif]

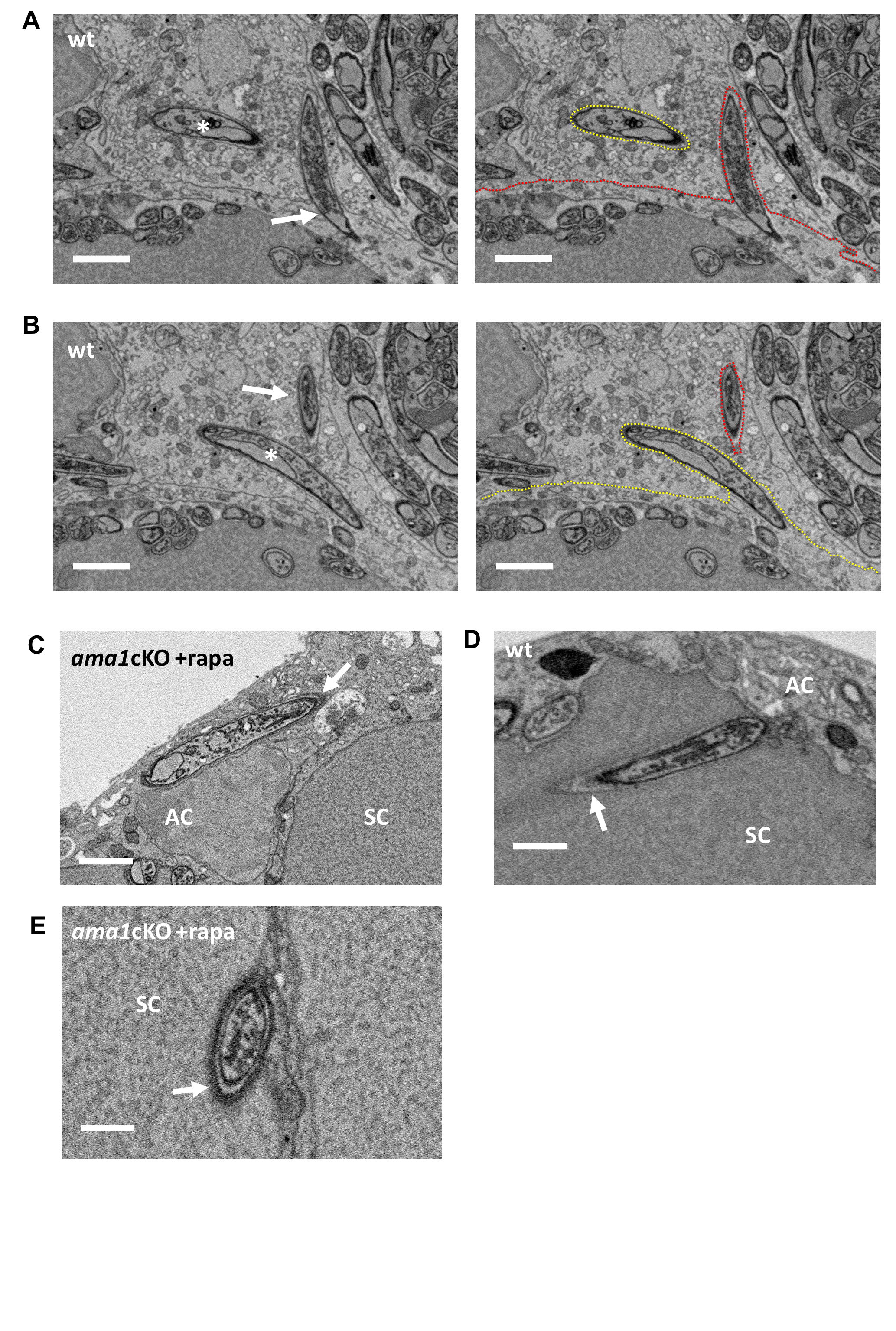

Supplement: S12 Fig — A-B. SBF-SEM sections from S3 Movie, showing WT sporozoites inside salivary gland acinar cells. The first section (A) shows a sporozoite partly surrounded by host cell membranes (arrow), highlighted in red in the right panel, and a second one seemingly contained inside a vacuole (asterisk), highlighted in yellow in the right panel. The second section (B) shows the same parasites in a different plane, revealing that the second sporozoite is in fact not enclosed in a vacuole but instead is interacting with invaginated host cell membranes (asterisk), highlighted in yellow in the right panel, while the first parasite now seems surrounded by a membrane (arrow), giving the false impression of being enclosed in a vacuole (highlighted in red in the right panel). Scale bars, 2 μm. C. SBF-SEM section showing an intracellular rapamycin-treated ama1cKO sporozoite surrounded by a cellular membrane (arrow). Scale bar, 2 μm. AC, acinar cell; SC, secretory cavity. D-E. SBF-SEM sections showing WT (D) and rapamycin-treated ama1cKO (E) sporozoites present inside secretory cavities (SC) and surrounded by cellular membranes (arrows). Scale bars, 1 μm. (TIF) [file ppat.1010643.s015.tif]

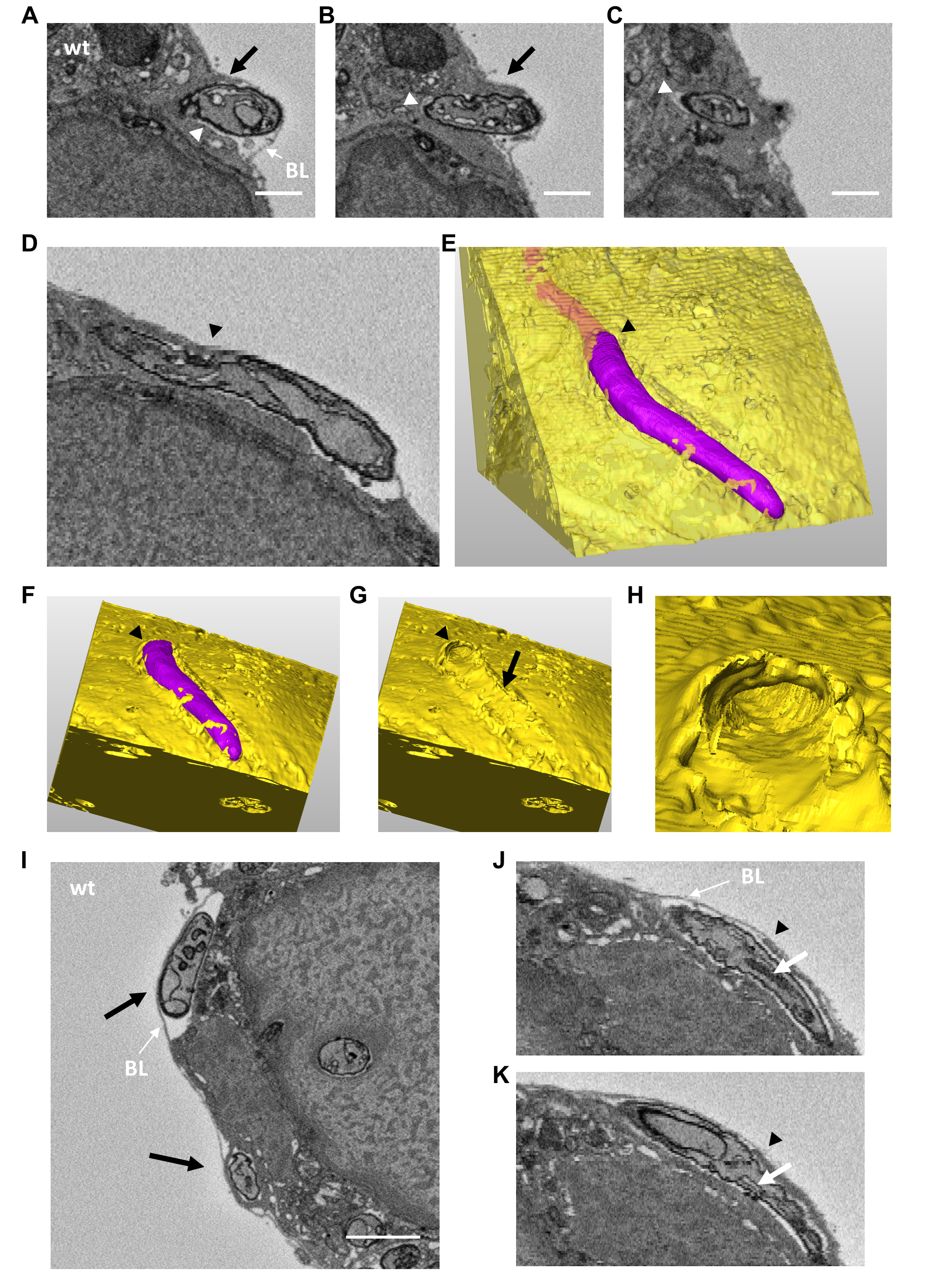

Supplement: S13 Fig — A-H. SBF-SEM images of an invading untreated ama1cKO sporozoite. Panels A-C show three XY sections of the invading parasite. The sporozoite is located underneath the basal lamina (BL), and enters the cell surrounded by a vacuole (white arrowhead). The entry site is marked by a black arrow. Scale bar, 1 μm. Panel D shows a virtual XZ section, illustrating that the sporozoite is penetrating tangentially into the acinar cell. The entry aperture is marked by a black arrowhead. Panels E-H show a volume segmentation of the parasite (in purple) invading the mosquito cell (in yellow). The entry site is marked by a black arrowhead. In G and H, only the cell surface is shown, revealing the imprinting of the extracellular portion of the sporozoite (black arrow). In H, the circular entry site is shown at higher magnification. I-K. SBF-SEM images of another invading untreated ama1cKO sporozoite. In I, a XY section cuts the invading parasite twice (black arrows), with the extracellular portion being positioned between the cell surface and the basal lamina (BL). Two virtual YZ sections are shown in J and K, illustrating that the sporozoite is penetrating tangentially into the acinar cell. The entry aperture is marked by a black arrowhead. A full rhoptry is visible in J and an empty one can be seen in K (arrows). (TIF) [file ppat.1010643.s016.tif]

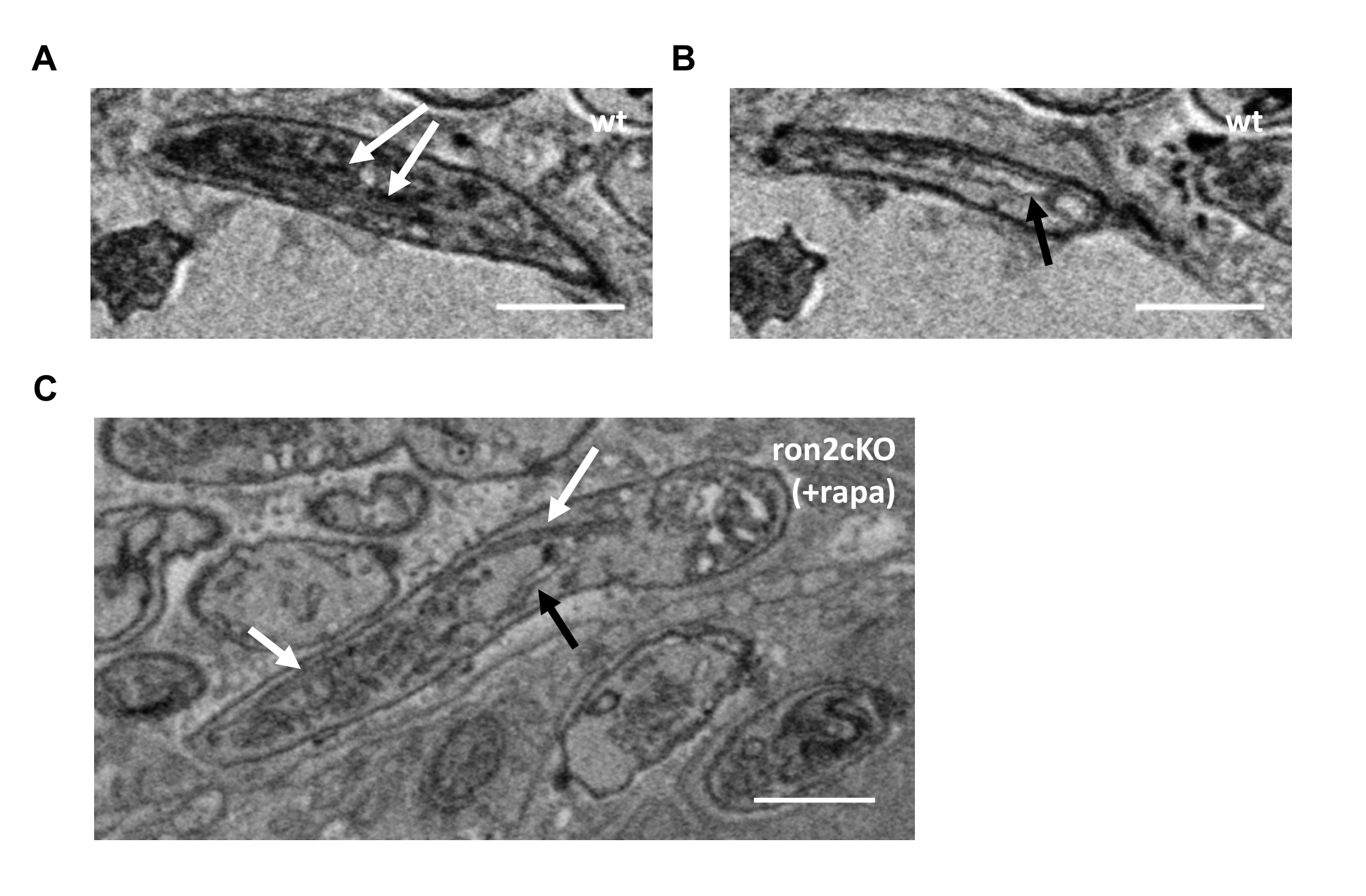

Supplement: S14 Fig — A-B. SBF-SEM sections of the apical end of an intracellular untreated (wt) ama1cKO sporozoite. In A, two full rhoptries are visible, indicated by white arrows. In B, a different section of the same parasite reveals an empty rhoptry (black arrow). C. SBF-SEM section of an intracellular rapamycin-treated ron2cKO sporozoite, showing two full rhoptries (white arrows) and one empty one (black arrow). Scale bars, 1 μm. (TIF) [file ppat.1010643.s017.tif]

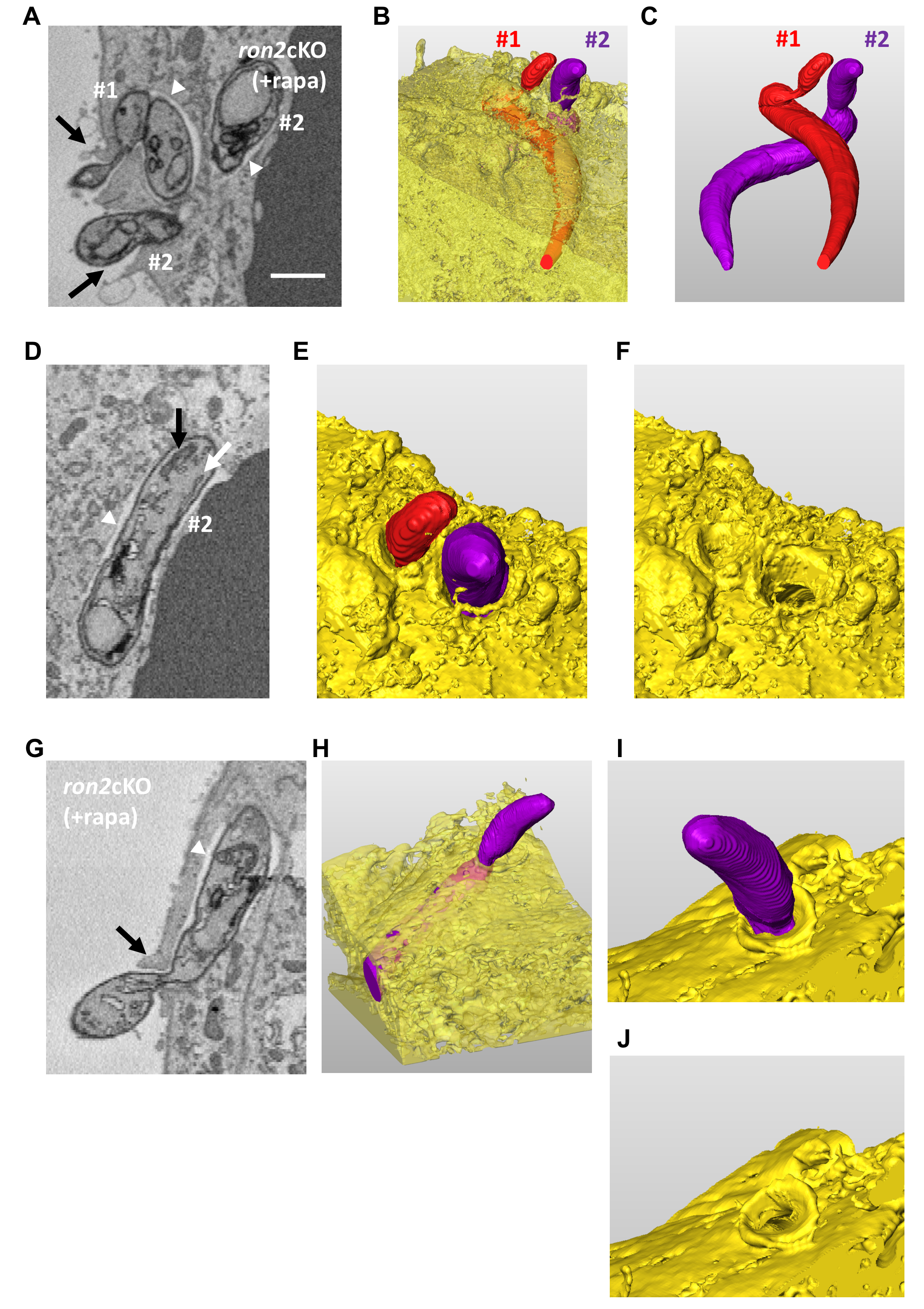

Supplement: S15 Fig — A-F. SBF-SEM images of two invading rapamycin-treated ron2cKO sporozoites. In A, the first sporozoite (labelled #1) is cut once, while the second one (#2) is cut twice. The entry sites are indicated by black arrows, and the vacuoles by white arrowheads. Scale bars, 1 μm. Panels B and C show volume segmentation images of the invading parasites (red and purple, respectively). The cell is colored in yellow. Panel D shows a virtual XZ section, showing the vacuole (white arrowhead), a full rhoptry (black arrow) and an empty vesicle (white arrow). G-J. SBF-SEM images of another rapamycin-treated ron2cKO sporozoites. In G, the entry site is indicated by a black arrow, and the vacuole by a white arrowhead. Panels H-J show volume segmentation images of the invading parasite (purple). The cell is colored in yellow. The entry site is shown at higher magnification in I and J, with or without displaying the sporozoite. (TIF) [file ppat.1010643.s018.tif]

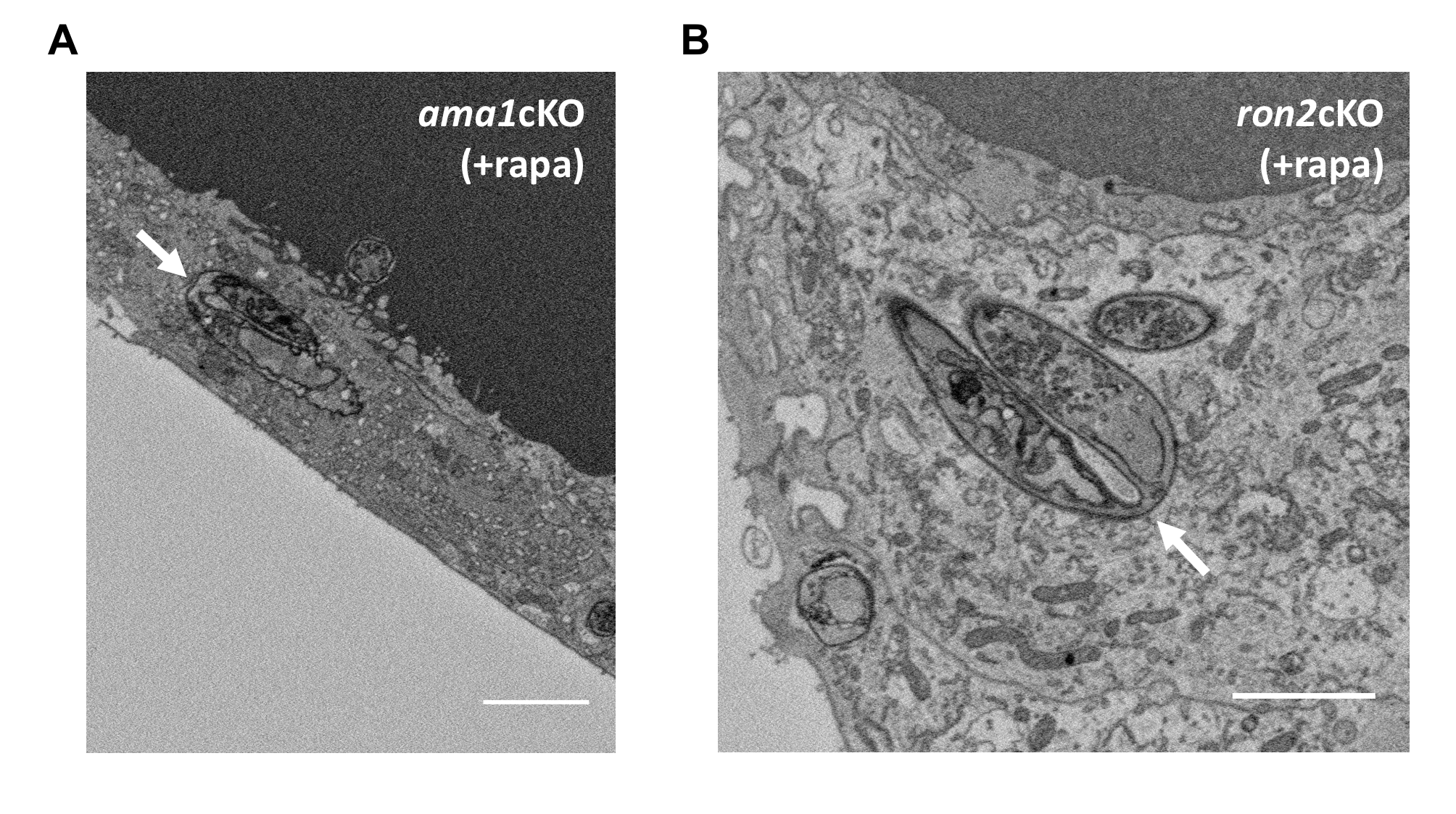

Supplement: S16 Fig — A-B. SBF-SEM sections of intracellular rapamycin-treated ama1cKO (A) and ron2cKO (B) sporozoites. Both parasites display a strong bending, with the hinge indicated by an arrow. Scale bars, 2 μm. (TIF) [file ppat.1010643.s019.tif]

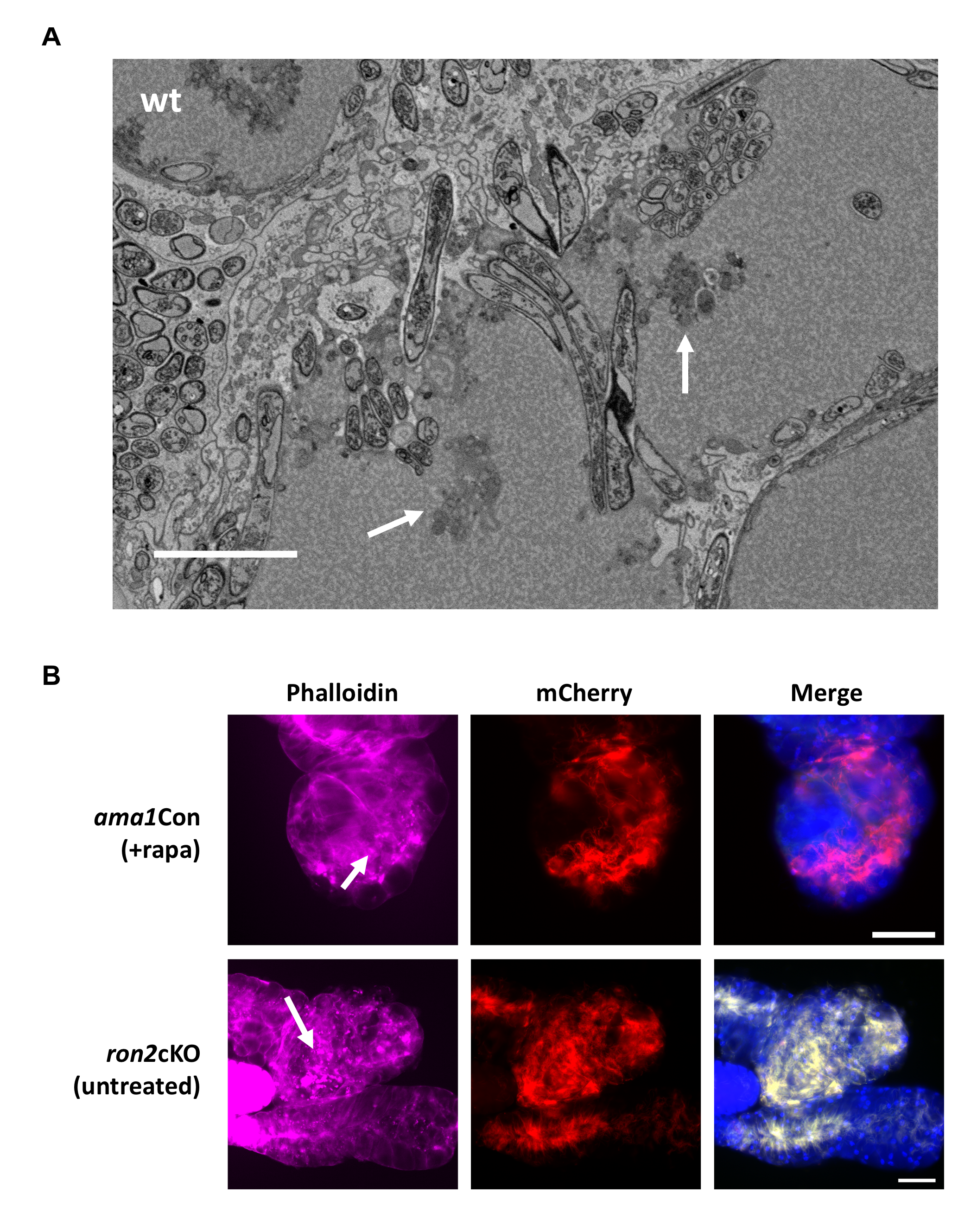

Supplement: S17 Fig — A. SBF-SEM section showing an alteration of the cellular interface with the secretory cavity at the point of entry of multiple WT sporozoites (asterisk). Intraluminal leakage of cytoplasmic material is indicated with an arrow. Scale bar, 5 μm. B. Fluorescence microscopy images of salivary gland distal lobes infected with rapamycin-treated ama1Con or untreated ron2cKO parasites. Samples were stained with Phalloidin-iFluor 647 (magenta) and Hoechst 77742 (Blue). The right panels show mCherry (red), GFP (green) and Hoechst (blue) merge images. In both cases, the heavy parasite load is associated with internal alterations of the phalloidin staining, but the basal border of the lobes is preserved. Scale bars, 50 μm. (TIF) [file ppat.1010643.s020.tif]

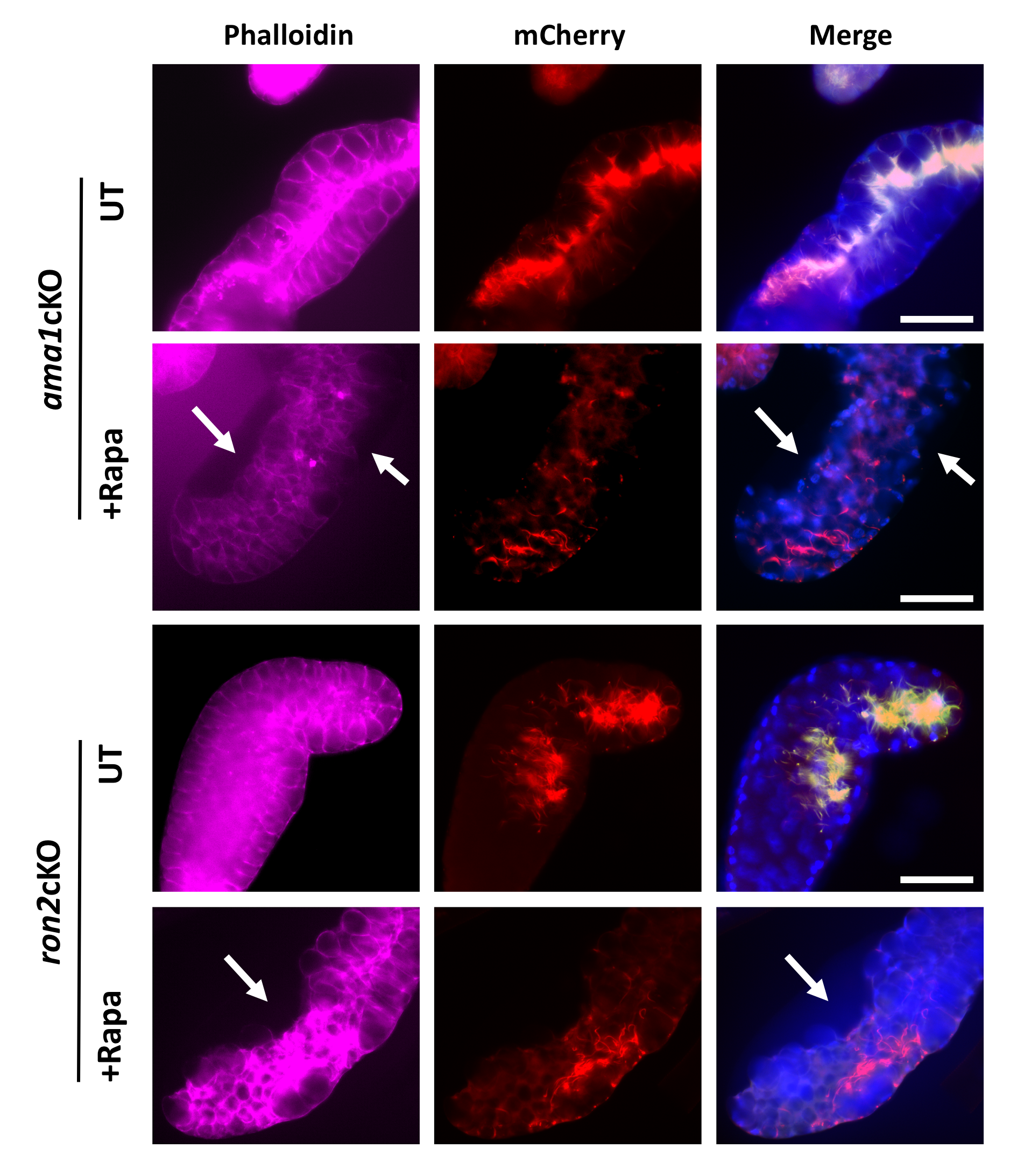

Supplement: S18 Fig — Representative fluorescence microscopy images of salivary gland lobes infected with untreated (UT) or rapamycin-treated (+Rapa) ama1cKO or ron2cKO parasites, day 16 post-infection. Samples were stained with Phalloidin-iFluor 647 (magenta) and Hoechst 77742 (Blue). The right panels show mCherry (red), GFP (green) and Hoechst (blue) merge images. Zones of retraction of the acinar epithelial cells are visible in the lobes infected with AMA1- and RON2-deficient sporozoites (arrows). Scale bars, 50 μm. (TIF) [file ppat.1010643.s021.tif]
