# Supplementary material for: The AMA1-RON complex drives Plasmodium sporozoite invasion in the mosquito and mammalian hosts
Source: PLoS Pathog. 2022 Jun 22;18(6):e1010643. doi: 10.1371/journal.ppat.1010643 (PMC9255738; doi:10.1371/journal.ppat.1010643)
Supplement: Attachment — Submitted filename: Response to reviewers.pdf [file ppat.1010643.s028.pdf]

PPATHOGENS-D-22-00054
Author's response (in blue; line numbers refer to the clean version)

Editors comments

As you will see, all three reviewers agree that this work resolves previous contradictions in the field and potentially provides important and exciting new insights into the role of AMA1 and the partner RON proteins in sporozoite biology. The indications that the AMA1 complex enables entry into mosquito salivary glands without cell damage is considered particularly significant. However, all the reviewers believe that more quantitative and detailed analysis of the epithelial damage described in Figure 6 is required to support your conclusions, and the nature of those parasites that survive (and invade hepatocytes) following gene disruption requires characterisation. A number of other concerns are also raised by the reviewers that are important to address.

We would like to thank the three reviewers for their positive and constructive comments. As detailed below in our point-by-point response, we have now addressed all the comments. In particular, we provide new volume electron microscopy data capturing sporozoite invasion events into salivary glands and providing more evidence of epithelial cell damage. The new data consolidate a model where sporozoites use the AMA1-RON complex to enter salivary glands through a junction and by forming a transient vacuole (new Fig 5 A-F and Fig S13-S14). Our data also indicate that in the absence of a functional AMA1-RON complex sporozoites are still capable of forming a junction, which however shows distinctive features as compared to WT parasites (new Fig 5 G-K and Fig S15-S16). As a result, sporozoite penetration is impaired, associated with collateral epithelial damage (new Fig6 and S18), possibly due to a sealing defect at the entry point. We have extensively modified the discussion to take into account these new results.

Part II – Major Issues: Key Experiments Required for Acceptance

Reviewer #1:
Figure 6 could be expanded by showing two images from EM and maybe enlargements from the fluorescent images. This would be helpful as Supplementary Figure 3B and 5B shows mostly disrupted salivary glands suggesting that the effect reported in Figure 6 might be due to damage inflicted during preparation. In this line, Supplementary Figure 11 shows intact glands and could be expanded by more examples.

In Fig S3B and S5B, salivary glands were collected and imaged without fixation, as the purpose of these experiments was only to analyze the GFP fluorescence of the parasites (excised versus non-excised). Under these conditions, salivary glands were prone to damage due to sample preparation. As shown in the revised Fig 6 using fixed samples, epithelial cell damage (as evidenced by retraction of the epithelium) was observed with both control (untreated) and rapamycin-treated parasites. However, the frequency of such damage was significantly higher with treated samples (around 50% as compared to 10-25%, new Fig6G-H), suggesting that invasion through dysfunctional junctions may lead to defects in membrane sealing and collateral cellular damage due to fragilization of the epithelium. In good agreement with this hypothesis, our new SBF-SEM data provide evidence of rupture of the basal lamina and in

some instances of the cell plasma membrane, associated with cell vacuolization in salivary glands infected with rapamycin-treated *ama1*cKO or *ron2*cKO parasites. These new results have been included in the revised manuscript in Fig 6 (panels C-F).

Reviewer #2:
1. Fig. 2B and 4B show a dramatic reduction in parasite growth following rapamycin treatment. But there appears to be some parasites present even on day 5. Are these a result of adaptation? The authors could continue selection of these parasites to see what if any adaptation occurs in the absence of these genes.

We genotyped *ama1*cKO blood stage parasites by PCR at day 2 and day 6 post-rapamycin treatment, confirming *ama1* gene excision. However, genotyping also revealed the persistence of non-excised parasites at both time points, which eventually outcompeted the excised population. These data are shown in the new Fig S4C, and discussed in the revised text (lines 178-181).

2. Fig 5 and 6 (and S11) show representative images. The authors should provide quantitative data to demonstrate the loss of epithelial integrity.

We have now extensively modified these figures. Fig 5 now shows sporozoite invasion events captured by SBF-SEM, while Fig 6 shows epithelial damage (including new SBF-SEM and fluorescence microscopy images). Epithelial cell damage (as evidenced by retraction of the epithelium) was observed with both control (untreated) and rapamycin-treated parasites. However, the frequency of such damage was significantly higher with treated samples (around 50% as compared to 10-25%), suggesting that invasion through dysfunctional junctions may lead to defects in membrane sealing and collateral cellular damage due to increased fragility of the epithelium. These quantitative data are now shown in the new Fig 6H, with statistical significance indicated in the figure legend.

3. If epithelial integrity is extensively compromised as the authors suggest, mosquito feeding behavior may be affected. The authors should test for this.

This is an interesting remark. Although we have not analyzed mosquito feeding behavior, it is indeed possible that damage inflicted to the glands could have additional detrimental effects on parasite transmission with the mutants (in addition to the reduction of parasite loads in the glands), including impaired feeding or reduced mosquito survival. We have included a comment on this point in the discussion (lines 615-616).

4. AMA1cKOrapa sporozoites infect hepatocytes in vitro to nearly 1/3 the levels of WT parasites. The authors suggest it could be adaptation. Could this be an in vitro artifact? The authors should perform in vivo infections both through mosquito bite as well as injection of sporozoites. This can highlight important roles during the transit from the skin to the liver.

Our data show that the deletion of *ama1* or *ron2*/*ron4* leads to a >10-fold reduction in salivary gland invasion, and a 3-6-fold reduction in HepG2 cell infection. These results are in total agreement with the data from Ishino's group for RON2, showing a 3-4-fold reduction of parasite loads *in vivo* in mice and *in vitro* in HepG2 cell cultures infected with RON2 knockdown

mutants (ref 14). Therefore we do not think that the *in vitro* data are an artefact. Rather, we hypothesize that different membrane properties probably impact differently the function of the junction during invasion of salivary glands versus hepatocytes (as discussed lines 653-657). It is possible that more severe defects would be observed under *in vivo* conditions, but in our hands the low numbers of AMA1- and RON-deficient sporozoites recovered from mosquito salivary glands precluded their analysis *in vivo* in mice. We added this point in the discussion (lines 649-651).

5. Can the authors test if AMA1 functions independent of RON2/RON4 (and vice versa) in KO sporozoites that invade hepatocytes?

This is difficult to answer. In our study we independently knocked-out *ama1*, *ron2* or *ron4*. The phenotypes with all three mutants are similar, with a 3-6 fold reduction in hepatocyte invasion *in vitro*, which is in line with the previous study of Ishino et al on RON2. Therefore, our data do not support that AMA1 and RONs play independent roles during sporozoite invasion of mosquito salivary glands or mammalian hepatocytes.

Reviewer #3: Major issues to address:
1. The controversy over the role of AMA-1 in hepatocyte invasion is primarily due to 2 papers from the Menard group, which used the Cre-Lox system to delete ama1. The disadvantage of this system is that Cre recombinase is driven by a stage-specific promoter, either csp or trap. Thus, gene deletion occurred in the mosquito, which does not give sufficient time for any residual protein to be completely degraded. In contrast, the DiCre system enabled these investigators to delete ama1, in the blood stages (after gametocyte formation) and gave ample time for any residual AMA1 to be degraded. Over time it has become clear to many sporozoite researchers that the Cre-Lox system developed by the Menard group is most informative for dissecting mid to late liver stage phenotypes and because of the confounding issue of residual protein, which is not always easy to observe by IFA, is not conclusive for proteins involved in sporozoite invasion of hepatocytes. It would be helpful to the field, if the authors could clarify why their system, but not the previous Cre-Lox system, was successful in demonstrating a role for AMA1 in sporozoites. Since the other previously published papers on AMA1 and sporozoites are supportive of the data shown here, a clarification of why the dissenting papers are likely wrong, further helps to unify the findings.

We thank the reviewer for this important comment. Indeed, due to the late timing of gene excision, the flp/FRT system used by the Ménard group was not suitable to detect a salivary gland invasion phenotype, and possibly was not sufficient to completely abrogate protein expression in sporozoites. This fundamental difference between the flp/FRT system and our DiCre approach likely explains the discrepancy between the studies. We have added an entire paragraph in the discussion section to clarify this point (lines 544-554).

2. The data shown in Figure 6 give rise to a novel and important hypothesis, namely that tight junction entry into mosquito salivary glands minimizes damage to the host. The confocal images suggest retraction of some epithelial cells but the damage is not clearly demonstrated. There is damage demonstrated in the one EM shown but that is n=1. Could they embed the glands and do straight-forward H&E staining to show damage to the basement membrane?

Perhaps incubating the salivary glands with high MW dextran conjugated to fluorescein would demonstrate holes in the glands entered by the cKO parasites? Additional data are needed to bolster what is the most novel and interesting finding of the paper.

We have performed additional SBF-SEM imaging at an earlier time point (day 15), and could capture invasion events showing WT or RON2-deficient sporozoites caught in the process of penetrating the glands (new Fig5, S13-S16, Movies 4-6). Our new SBF-SEM data also document rupture of the basal lamina and in some instances of the cell plasma membrane, associated with cell vacuolization in salivary glands infected with rapamycin-treated *ama1*cKO or *ron2*cKO parasites. These new results have been included in the revised manuscript in Fig 6 (panels C-F). Epithelial cell damage (as evidenced by retraction of the epithelium) was observed with both control (untreated) and rapamycin-treated parasites. However, the frequency of such damage was significantly higher with treated samples (around 50% as compared to 10-25%), suggesting that invasion through dysfunctional junctions may lead to defects in membrane sealing and collateral cellular damage due to increased fragility of the epithelium. These quantitative data are now shown in the new Fig 6H, with statistical significance indicated in the figure legend.

Part III – Minor Issues: Editorial and Data Presentation Modifications

Reviewer #1:
Please add references to the different systems described in lines 94-95. Maybe mention that they were first introduced to apicomplexan research in Toxoplasma gondii
We have added references (including to the pioneering work done in *Toxoplasma*), as requested (lines 94-97).

Figure 1 "hoechst" should be "Hoechst"
This has been modified.

In my view fluorescent images are best presented as black on white and not color on black, which gives very bad contrast even on the screen. (also for other figures showing sporozoites)

We agree with the reviewer that black on white images with higher contrast are sometimes useful, especially for localization studies. However, we preferred to keep the colored images as our purpose was to illustrate the GFP/mCherry fluorescence of the parasites (GFP+/non-excised versus GFP-/excised). Nevertheless, we increased the contrast in these figures to facilitate viewing.

I am not sure whether the writing of UT and rapa in the exponent of the parasite line name is the best way of writing but have no immediate other easy to read alternative.

We thank the reviewer for this comment. We agree that writing UT and rapa in the exponent was not easy to read. Therefore we changed the writing throughout the manuscript and figures, and now simply refer to untreated or rapamycin-treated parasites.

Line 171: please state in text how you reused the ama1Conrapa parasites, negative selection and marker recycling?

Rapamycin treatment of the *ama1*Con parasites was sufficient to delete the GFP-hDHFR resistance cassette in addition to leaving a residual single LoxN site downstream of *ama1* gene, without the need for negative selection. This has been clarified in the revised text (lines 167-169).

Figure 2B: could you take parasites from day 4 or 5 and look by PCR if AMA1 is indeed lost in at least a subset of them?

We genotyped *ama1*cKO blood stage parasites by PCR at day 2 and day 6 post-rapamycin treatment, confirming *ama1* gene excision. However, genotyping also revealed the persistence of non-excised parasites at both time points, which eventually outcompeted the excised population. These data are shown in the new Fig S4C, and discussed in the revised text (lines 178-181).

Line 190: there are not 4 but 2 lines treated differently, please rephrase

We have modified the sentence, as suggested (line 212 in the revised manuscript).

L 251.Over 90% looks more like over 95% in graph

We have modified the sentence, as suggested (line 251 in the revised manuscript).

L 259: Please specify which sporozoites you used, from hemolymph or salivary glands?

We used salivary gland sporozoites. This is now specified in the revised text (line 258).

Panels in Figure 5C-H could be increased in size and shown as doubles with specific features highlighted with colours in one of the copies.

Fig 5 has been changed to show invasion events. Panels from former Fig 5 are now shown in revised Fig S12 and S17, and have been increased in size. As suggested, we have included panels showing host cell membranes highlighted with different colors (new Fig S12A-B).

L863+953: references incomplete

We have corrected the references.

Movie 3 appears very pixilated. If somehow possible this beautiful dataset should be improved.

We apologize for the complete loss of resolution due to overt compression during format conversion. We now provide movies (including Movie 3) at high resolution, in mp4 format.

Reviewer #2:
1. The authors argue that deletion of AMA1 3'UTR is not sufficient to abrogate protein expression and could explain the lack of phenotype observed in prior Pb studies. However, the presence of the AT-rich 3'HR that follows the hDHFR cassette in the ama1dUTR construct (Fig.S1) may be sufficient to provide transcript stability. This is in contrast to the previous study

where sequences from the plasmid backbone replaces the 3'UTR and are likely to not function as viable 3'UTR in the parasite.

We agree with the reviewer that differences in the downstream sequence could explain the discrepancy between our study and the previous one, in terms of AMA1 protein expression following 3'UTR excision. We have added this point in the revised discussion (lines 556-560).

2. Fig 1: The flow panel and the labels are too small to discern. Requires bigger labels.

The flow cytometry panels have now been moved to Fig S1C and Fig S2C, with increased size and bigger labels.

3. Fig.S5A: If this is representative figure, it appears there may be significantly fewer oocysts in ama1cKOrapa compared to untreated. The lower number of total midgut sporozoites seen in Fig2C may reflect this lower oocyst densities.

We thank the reviewer for pointing at this figure. We have not observed any obvious reduction in the number of oocyst in mosquitoes infected with rapamycin-treated *ama1*cKO parasites as compared to the untreated condition. Therefore we replaced this figure with a more representative one (new Fig S5A).

4. Fig 3C: There appears to be a small number (5-10%) of "excised" parasites in the un-treated groups, how?

The % of mCherry-positive GFP-negative ("excised") EEFs was determined by FACS, and we always observed a background of GFP-negative cells among mCherry+ cells, usually around 2-3% but up to 5-7% in one experiment (see Table S3 for the raw data). We don't have a clear explanation for this phenomenon, but it might be due to autofluorescence of some cells in the red channel, or reflect the loss of GFP due to acidification of the vacuole in a minor proportion of the parasites, rather than actual gene excision. In any case, this is a marginal observation that does not affect the conclusions drawn from the data in Fig 3C.

Reviewer #3: Minor issues:
1. In Figure 1 the flow plots could be clarified by stating that the vast majority of cells are uninfected RBCs. Also why are there no mCherry cells in the untreated DeltaUTR and Con parasites? Lastly, why are the percentage of GFP parasites so much lower than the percentage of mCherry parasites after rapamycin treatment – I would have expected the opposite.

The flow plots have been moved to supporting information as Fig S1C and S2C in the revised manuscript. We have modified the labels to better illustrate mCherry+GFP+ (non excised) and mCherry+GFP- (excised) parasites, and indicate in the plots the non-infected RBC population. Fig S1C and S2C clearly show that in the untreated DeltaUTR and Con parasites the infected RBCs are mCherry-positive an GFP-positive (the mCherry-negative GFP-negative cells are non-infected RBCs). The P. berghei DiCre line contains a mCherry cassette that is constitutively expressed and is not affected by the addition of rapamycin. Therefore all the parasites should be mCherry-positive, irrespective of the treatment condition. In all the parasite lines generated in this study, we added a GFP cassette upstream or downstream of the target gene.

Upon addition of rapamycin the GFP cassette is excised, together with the gene, explaining why the % of GFP-positive parasites are very low after rapamycin treatment.

2. Figures 2J, 3B, 3C, and 4J, need denominators, which can be included in the legend. It would be important to know for example whether the untreated controls in Fig 3B are approximately similar. If the ama1cKO untreated parasites are significantly less robust than the ama1Con untreated parasites one could interpret the rapamycin data somewhat differently. Overall the interpretation of the data by the reader would be aided by some absolute numbers.

All the raw data (absolute numbers), normalized results and statistical analysis are provided in Table S3. It was difficult to compare all parasites at the same time due to limited numbers of mosquitoes. However, there are 2 experiments (labelled #3 and #10 in Table S3) where untreated *ama1*cKO and *ama1*Con were analyzed in parallel, showing no defect in the cKO line. This was expected since in this line, the *ama1* gene is intact before addition of rapamycin.

3. Line 273, the enrichment of unexcised ama1cKOrapa, sporozoites is small (but looks real). Might be more accurate to describe it as a "small enrichment" as it looks like its between 5-7%.

We changed the text, as suggested (line 300 in the revised manuscript).

4. Line 308, RFP needs defining.

We changed the text, as suggested (line 314 in the revised manuscript).

5. Figure 5 – the still photographs shown are difficult to see the host versus parasite membranes. Perhaps these could be labeled better. Also these data are largely in line with previous studies in which sporozoite invasion of salivary glands was studied and this could be more clearly stated in the discussion.

Panels from former Fig 5 are now shown in revised Fig S12 and S17, and have been increased in size. As suggested, we have included panels showing host cell membranes highlighted with different colors (new Fig S12A-B). We have extensively modified the discussion to take into consideration the new SBF-SEM data shown in the new Fig 5, S13-S17. These new data consolidate a model where sporozoites use the AMA1-RON complex to enter salivary glands through a junction and by forming a transient vacuole. Formation of transient vacuoles during invasion of salivary glands has been documented in a previous study (Pimenta et al 1994). Our work provides mechanistic insights by showing the role of the AMA1-RON complex in this process. In the absence of a functional complex, sporozoite penetration is impaired, associated with collateral epithelial damage, possibly due to a sealing defect at the entry point.
